# Notes and News

**Published:** 1891-06-20

**Authors:** 


### Notes and News.

Collected and Communicated.

We regret to announce the death from influenza of Dr. Henry Gawen Sutton, one of the physicians of the London Hospital, and for a quarter of a century Medical Officer of Health for St. Leonard's, Shoreditch. Dr. Sutton was the author of several works on sanitary questions, and on the subject of epidemics was regarded as an authority.

An anonymous donor has given the county of Surrey a valuable gift in the pretty Convalescent Home at Seaford. Neither money nor pains have been spared in the building of this much-needed institution, which was began three years ago, and has cost, including about £10,000 set aside as endowment, no less than £25,000. The Home will accommodate fifty patients, and the building is so constructed as to allow its being made larger when necessary. The Committee are about to make an appeal for subscriptions in aid of both the endowment and maintenance funds, which should meet with a cordial response by the people of the county, who can show their sense of gratitude to their anonymous benefactor by backing up the efforts he has made to establish this Home and to make it a permanent institution.

The new Convalescent Home of the Chelsea Hospital for Women, which has been erected at St. Leonards, was opened by the Countess Cadogan on the 3rd inst. With the generous assistance so far accorded to the Home, the Committee have acquired the freehold of the excellent site, besides erecting the Home at a cost for the building alone of £3,380. The foundation-stone was laid in July last by Dr. Aveling, who, at the opening ceremony, gave some details of the founding of the Chelsea Hospital twenty years ago, and of the prosperity which had attended its growth ever since. An address was presented to Lady Cadogan, who then, with a golden key, unlocked the door and declared the Home to be open. The party, accompanied by the Ladies' Committee, were shown over the Home by the Matron, the arrangements of which were much admired.

A quarterly general Court of Governors of the London Hospital was held on the 3rd inst. Mr. T. H. Burton presided, and in moving the adoption of the report announced that the Duke of Cambridge had kindly consented to open the new buildings on the 26th inst., and that the Archbishop of Canterbury had also promised to attend on the occasion. The motion was seconded by Mr. Morris Abrahams, and Dr. B. Fenwick proposed the following amendment: "That this Court, while adopting the report, expresses its strong disapproval of the action taken by the Committee's officials of the London Hospital in petitioning the Board of Trade to refuse a small and frequently granted privilege to the Royal British Nurses' Association, namely, to omit the word 'limited' after its name, if the Association was registered as a limited liability company." Mr. C. C. Bailey seconded the amendment, which was defeated by 23 votes to 9, and a further amendment by Dr. B. Fenwick having been negatived, the report of the Committee was agreed to.

On Sunday last the West-end United Friendly Temperance and Trade Societies held their seventh annual demonstration and church parade on behalf of the funds of the Royal Free Hospital, Gray's Inn Road. The procession was formed on the Victoria Embankment and marched to the church of the Holy Trinity, Gray's Inn Road, where a sermon was preached by the Rev. Frederick Thorne. As it is well known that the Royal Free Hospital is greatly in want of funds, a substantial sum should be realised by the demonstration.

The private house, giving accommodation for two beds, which was opened in 1888 as a cottage hospital for Gorleston, was soon found to be quite inadequate to meet the demands made upon it. The Board of Governors, therefore, determined to build a specially designed hospital, for which the Chairman gave a piece of land as a site. Owing to a scarcity of funds, only a portion of the building, containing four beds and other rooms, has been erected. Fifty-four in-patients and 878 out-patients have been treated in this little hospital since its foundation. It is supported by voluntary subscriptions, and meets a great want among the fishermen and inhabitants of the neighbourhood. The greatest economy has been exercised in the management, the average expenditure, including all charges, being only £200 per annum. Unfortunately, the income is still smaller, and additional annual subscribers are greatly wanted in order to clear off the debt still remaining on the building, and also to assist the working expenses.

The annual court of the governors of the Hospital for Consumption, Brompton, was held on May 28th, under the presidency of the Earl of Derby, K.G. The Secretary, Mr. Dobbin, read the fiftieth annual report, which showed that of 1,845 in-patients during 1890, 1,426 were discharged, many greatly benefited, while there were 239 deaths. The daily average number of beds occupied was unusually low, owing to the fact that the south wing was unoccupied for about six months on account of the needful sanitary improvements and other works. The receipts from all sources amounted to £32,533, including a large amount in legacies. The expenditure was £24,495; 479 in-patients were sent to the Seaside Convalescent Home at Sandgate for a month, the cost of their maintenance there, railway fares, &c., being entirely borne by the hospital. This year is the jubilee of the hospital's existence, and the committee are anxious that it should be marked by unusual financial prosperity. The report was adopted on the motion of the Chairman, seconded by Mr. A. Warre, and, after some formal appointments, the proceedings closed.

Lord Robartes, in presiding at the annual meeting of the Hospital for Epilepsy and Paralysis, Regent's Park, on Saturday, at the Marlborough Rooms, alluded to the fact that an anonymous gift of £1,200 had enabled the Committee to purchase the unexpired ten years of the lease of Winterton House, where the hospital for so many years has found a home, and dwelt emphatically on the point that, sooner or later, a new and larger hospital must be built. No site could be better than the one at present occupied, and he regretted to learn that there was much doubt as to the continuance, after the ten years had expired, of the hospital in its present healthy and delightful situation. Another so sanitary and pleasant could not easily be found.——The long-standing debt has now been completely extinguished, thanks to the untiring energies of the Secretary, Mr. Howgrave Graham, and we are sure that the friends of the hospital will aid him in preventing the institution, which has with infinite labour been dragged to the top of the hill, from gliding back to the marshy land of debt below. What is wanted are new annual subscribers and subscribers towards the building fund of £20,000, which the Committee wish to raise for the new hospital.

